# MuvB: A Key to Cell Cycle Control in Ovarian Cancer

**DOI:** 10.3389/fonc.2018.00223

**Published:** 2018-06-11

**Authors:** Audra N. Iness, Larisa Litovchick

**Affiliations:** Division of Hematology, Oncology and Palliative Care, Massey Cancer Center, Virginia Commonwealth University, Richmond, VA, United States

**Keywords:** p130, B-Myb, DYRK1A, protein complex, transcription, cell cycle

## Abstract

Cancer cells are characterized by uncontrolled proliferation, whereas the ability to enter quiescence or dormancy is important for cancer cell survival and disease recurrence. Therefore, understanding the mechanisms regulating cell cycle progression and exit is essential for improving patient outcomes. The MuvB complex of five proteins (LIN9, LIN37, LIN52, RBBP4, and LIN54), also known as LINC (LIN complex), is important for coordinated cell cycle gene expression. By participating in the formation of three distinct transcriptional regulatory complexes, including DREAM (DP, RB-like, E2F, and MuvB), MMB (Myb-MuvB), and FoxM1–MuvB, MuvB represents a unique regulator mediating either transcriptional activation (during S–G2 phases) or repression (during quiescence). With no known enzymatic activities in any of the MuvB-associated complexes, studies have focused on the therapeutic potential of protein kinases responsible for initiating DREAM assembly or downstream enzymatic targets of MMB. Furthermore, the mechanisms governing the formation and activity of each complex (DREAM, MMB, or FoxM1–MuvB) may have important consequences for therapeutic response. The MMB complex is associated with prognostic markers of aggressiveness in several cancers, whereas the DREAM complex is tied to disease recurrence through its role in maintaining quiescence. Here, we review recent developments in our understanding of MuvB function in the context of cancer. We specifically highlight the rationale for additional investigation of MuvB in high-grade serous ovarian cancer and the need for further translational research.

## MuvB Regulates Cell Cycle Gene Activation and Repression

Although unrestricted cell proliferation is one of the characteristics of cancer, malignant cells can enter a reversible quiescent state, enabling them to escape from treatments targeting rapidly dividing cells (1, 2). Understanding these processes is especially important in high-grade serous ovarian cancer (HGSOC) because of high rates of treatment resistance and recurrence. Retinoblastoma (Rb) family proteins, including pRb (Rb protein encoded by the *RB1* tumor suppressor gene), p107 (*RBL1*), and p130 (*RBL2*), are essential for entry into quiescence in mammalian cells (3, 4). pRb, p107, and p130 are also known as “pocket proteins” because they bind E2F transcription factors that regulate cell cycle-dependent genes using a conserved “pocket” domain (5). While the tumor suppressor role of pRb is well established, the roles of p107 and p130 in cancer are not fully understood. However, Rb-like proteins (but not pRb) can recruit the evolutionarily conserved DNA-binding protein complex MuvB to regulate gene expression. Recent studies reveal that through interaction with MuvB, p130, and p107 could play a unique and significant role in determining cancer aggressiveness and response to therapy.

**Figure 1:**
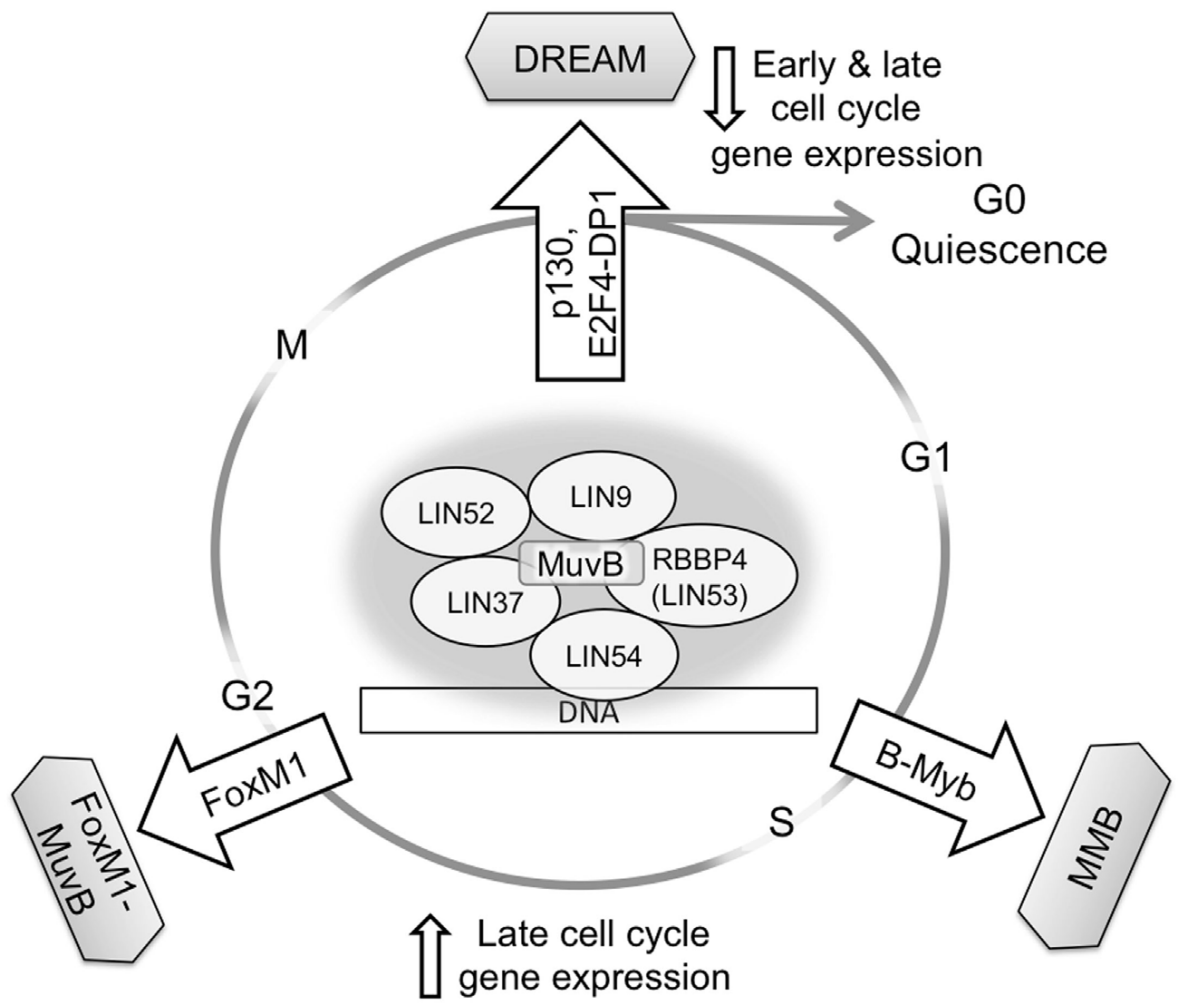
MuvB contributes to gene regulation throughout the cell cycle. MuvB binds p130/p107 and E2F4-DP1 in G0/G1 to form the DREAM complex and repress both early and late cell cycle genes. Upon cell cycle re-entry and during the S phase, MuvB binds B-Myb, forming MMB for expression of early cell cycle genes. The interaction between B-Myb and MuvB is important for recruiting FoxM1 for late cell cycle gene expression and subsequent mitosis.

Structurally related MuvB complexes including proteins encoded by the *LIN9, LIN37, LIN52, LIN54*, and *RBBP4* genes, or their orthologs, have been shown to regulate gene expression in different organisms including *C. elegans, Drosophila*, and *Homo sapiens* (6–9). In mammalian cells, MuvB participates in both repressor and activator gene regulatory complexes by alternating its binding partners at different points in the cell cycle. In G0/G1, MuvB is a component of the DREAM complex, which functions to repress gene expression for entering and maintaining quiescence. DREAM consists of p130, E2F4, and DP1 bound to MuvB, and its assembly requires phosphorylation of the LIN52 subunit of MuvB by dual-specificity tyrosine phosphorylation-regulated kinase (DYRK1A) (8, 10). DREAM disassembly occurs during the G1/S transition when cyclin-dependent kinases CDK4 and CDK2 phosphorylate p130 and MuvB subunits (8, 11–13). MuvB then dissociates from p130 and E2F4, leading to transcription of early cell cycle genes, including B-Myb and FoxM1 transcription factors. B-Myb recruits MuvB during the S phase, forming the MMB complex that binds to promoters of late cell cycle genes (10, 13–15). Furthermore, upon proteasomal degradation of B-Myb in S/G2, MuvB mediates timely recruitment of FoxM1 transcription factor to promoters of genes required for mitosis (15, 16). Therefore, by sequential association with three different DNA-binding transcription factors (E2F4, B-Myb, and FoxM1), MuvB coordinates cell cycle gene expression from quiescence through mitosis (Figure 1) (17, 18). This unique function of MuvB is central to maintaining cell cycle regulation and appropriate responses to environmental stimuli. The degree of MuvB participation in quiescence-related (DREAM) or proliferation-related (MMB or FoxM1–MuvB) complexes could be an important factor in cancer biology and therapeutic response.

**Figure 2:**
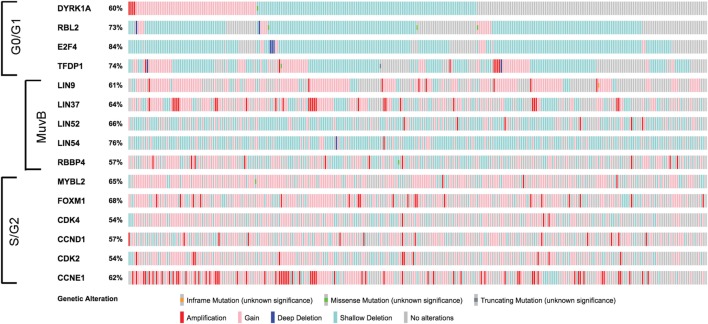
Alterations in genes involved in MuvB complex function. Figure shows summary of copy number alteration and mutation data from high-grade ovarian carcinoma samples (*N* = 316) visualized using cBio.org resource (19, 23). Note that the factors required for the G0/G1 function of the MuvB more frequently undergo genetic losses (blue color), whereas regions encoding genes associated with MuvB in S/G2 are frequently gained (pink) or amplified (red).

High-grade serous ovarian cancer is the most common of the epithelial malignancies in this disease site. Analysis of HGSOC data from The Cancer Genome Atlas (TCGA) reveals widespread variable genetic alterations of the factors involved in MuvB function (Figure 2) (19). Interestingly, genes encoding different MuvB subunits appear to be targeted both by gene copy number losses (LIN52 and LIN54) or gains (LIN9 and LIN37). MuvB’s involvement in complexes with different functions makes it challenging to parse out the contributions of individual proteins without understanding their exact roles in the context of each complex. Unlike pRb, mutations targeting p130 or p107 in cancer are rare (20–22). However, perturbations in DREAM activity could occur through its altered formation (e.g., aberrant activation of CDKs, inhibition of DYRK1A, or availability of MuvB components).

## Structural and Functional Studies of MuvB Subunits Reveal their Unique Roles

Since the discovery of mammalian DREAM in 2007, the structure and specific functions of the MuvB subunits are now beginning to emerge (24). Histone-binding protein RBBP4 (alias RbAp48) has been extensively characterized for its involvement in various chromatin-modifying complexes (25–27). Although there is no direct evidence of interaction between DREAM and any chromatin co-repressor complexes, RBBP4 likely serves as an adaptor to recruit such complexes to DREAM-regulated promoters. A recent study of mouse fibroblasts devoid of MuvB subunit LIN37 found that although the remaining subunits were able to assemble a DREAM-like DNA-binding complex, its repressor function was lost (28). Interestingly, the MMB-mediated transcription was not affected, suggesting that LIN37 specifically contributes to repressor role of MuvB. The smallest (116 a.a.) MuvB subunit LIN52 plays a key role in DREAM formation by direct interaction with p130 or p107. This interaction requires phosphorylation of serine 28 in LIN52 by DYRK1A (10, 11). Importantly, a different region in LIN52 is also essential for MMB complex formation. Therefore, LIN52 phosphorylation status and availability could impact the function of both complexes (11). Studies in cell lines and mouse genetic models reveal the importance of another MuvB subunit, LIN9, for both cell proliferation and tumor suppression, emphasizing its structural role in both DREAM and MMB (11, 29–31). Recent work also implicated LIN9 in direct binding with FoxM1 for the formation of FoxM1–MuvB complex required for mitotic gene expression (32). Interestingly, while MuvB associates with DNA-binding transcription factors to achieve target gene specificity, it also possesses intrinsic DNA-binding activity through MuvB subunit LIN54 (33). LIN54 recognizes specific DNA sequences called cell cycle homology regions (CHR), and mutations disrupting the LIN54–DNA interface abolish the recruitment of MuvB to promoters harboring the CHR elements (16, 34). Many mitotic genes contain CHR elements required for their expression, consistent with finding that loss of LIN54 results in cell cycle arrest and mitotic defects (16, 33, 35, 36). Together, these findings characterize the contributions of the individual subunits that can, in part, explain the multifunctional nature of the MuvB complex.

## MuvB Function is Influenced by Major Tumor Suppressor Pathways

Discovery of mammalian MuvB complex further clarified the overlapping and unique roles of the Rb family members in cell cycle control. While pRb itself does not interact with MuvB directly, formation of DREAM appears to be the major role of the other pocket proteins, p130 and p107 *in vivo* (8, 37). Previous studies demonstrated that inactivation of all three pocket proteins (pRb, p107, and p130) in mouse fibroblasts is necessary to block entry into quiescence (3, 38). Similarly, fibroblasts lacking MuvB subunit LIN37, or cells defective in MuvB-pocket protein interaction, are able to arrest in G0/G1 despite de-repression of DREAM target genes and aberrant formation of the proliferation-related MMB complex under the conditions of G0/G1 arrest (28, 37). However, depletion of pRb resulted in escape from G0/G1 arrest in LIN37 knockout cells (28). MuvB therefore becomes an essential regulator of the cell cycle and guardian of quiescence in the absence of functional pRb. Notably, copy number losses or mutations in the *RB1* gene (encoding pRb) are present in 67% of TCGA HGSOC samples.

In addition to cooperating with pRb for cell cycle exit, MuvB is functionally linked with p53. Activation of p53 in response to environmental stimuli, such as DNA damage, results in replacement of MMB with DREAM through a p21-dependent pathway (39–41). This switch is required for global cell cycle gene repression. Frequent mutations of the *TP53* gene in cancer (notably in 96% of HGSOC tumors) could lead to de-repression of oncogenic DREAM target genes, such as *Survivin* (*BIRC5*), *CDC25C*, and *PLK1* (19, 40, 42, 43). Therefore, deregulation of the p53–p21–MuvB pathway could have important implications for clinical outcomes in cancer. Indeed, in p53-mutant breast cancer cells, MuvB failed to dissociate from B-Myb (MMB complex) and bind p130/E2F4 upon DNA damage to form DREAM (44). Similarly, doxorubicin treatment of HCT116 colon carcinoma cells led to an increase in the population of G2/M cells and mRNA levels of late cell cycle genes when p53 was inhibited (40, 43). Basal MMB was also more abundant in p53-mutant hepatocellular carcinoma (HCC) cells versus those with wild-type p53. Whereas DREAM assembly was increased with doxorubicin treatment in p53 wild-type cells, MMB complex formation was paradoxically increased upon treatment of p53 null cells (45). Importantly, MMB formation was essential for the survival of p53 null HCC cells after doxorubicin-induced DNA damage, suggesting that inactivation of DREAM and aberrant formation of MMB contributes to chemoresistance of cancers with functional loss of p53, including HGSOC.

Oncogenic human papilloma viruses, such as HPV16 and HPV18, are known to inactivate pRb and p53 pathways through actions of viral proteins E7 and E6, respectively (46, 47). The structure of the LIN52–p107 complex revealed that E7 protein disrupts the DREAM complex by competing with MuvB subunit LIN52 for direct binding to p107/p130 (11). Indeed, LIN52 binds to a cleft in p130 or p107 bound by the LxCxExL motif present in HPV E7 and other oncogenic viral proteins, suggesting that displacement of the MuvB from DREAM could be essential for viral genome replication. In cancer cells expressing oncogenic HPV E7, MuvB is predominantly recruited to the MMB complex and contributes to proliferation of these cells (48–50). Interestingly, expression of E7 can impair the p53-dependent cell cycle checkpoint, independently of E6-mediated p53 degradation, by blocking p53-induced downregulation of DREAM target genes (51). These findings demonstrate the importance of the p53–p21–MuvB pathway for maintaining the checkpoint function of p53, regulation of gene expression, and cell cycle arrest that is often altered in cancer (52).

## MuvB Involvement in Cancer

The significance of MuvB subunit expression in human cancers has not been extensively studied, and MuvB is mostly linked to prognosis through association with B-Myb. Both amplification of the 20q13 *MYBL2* locus (encoding B-Myb) and over-expression of MMB target genes are associated with aberrant cell proliferation, cell cycle deregulation, and poor prognosis in many cancers including breast, liver, and ovarian (45, 53, 54). In biochemical studies of HCC tumor-derived tissues, high LIN9–B-Myb (MMB) and low LIN9–p130 (DREAM) complex formation was associated with poor overall survival, despite no significant difference in LIN9 levels (45). These findings were independently corroborated in a bioinformatics study of HCC data from TCGA showing a significant correlation between elevated expression of *MYBL2, LIN9, LIN52*, or *FOXM1* and poor overall survival (55).

A recent study using a *K-Ras^G12D^;p53^null^* mouse model of lung cancer revealed an important role for MMB in tumorigenesis, whereby a conditional deletion of *B-Myb* or *Lin9* significantly suppressed tumor formation (56). This study also demonstrated that MMB target gene *KIF23* (*MKLP1*) was required for lung tumor formation and represents a potentially druggable MMB target. Investigation of MuvB, B-Myb, and FOXM1 targets in breast cancer cells yielded further ties to MMB-regulated kinesins, whereby inhibition of two targets (KIF23 and PRC1) significantly reduced MDA-MB-231 cell proliferation. Analysis of the TCGA breast cancer data revealed correlations between high expression of mitotic kinesins and poor outcomes, suggesting that these MMB-regulated genes could serve as a prognostic signature or therapeutic targets (57). Furthermore, several MMB downstream targets are included in a chromosomal instability signature, used to predict clinical outcomes in multiple cancer types (58, 59).

Whereas high MMB levels are associated with a poor prognosis in many cancers, DREAM could contribute to cancer recurrence by promoting cancer cell survival under stressful conditions. In gastrointestinal stromal tumors (GIST), the DREAM complex has been implicated in imatinib mesylate resistance by promoting entry into quiescence (60, 61). Depletion of LIN52, or simultaneous knockdown of both E2F4 and LIN54, significantly enhanced imatinib-induced GIST cell apoptosis when compared with drug treatment alone. Pharmacological inhibition of DYRK1A also significantly increased imatinib-induced GIST apoptosis. Therefore, modulating DREAM formation through DYRK1A kinase activity is a potential therapeutic angle.

## MuvB in Ovarian Cancer

The cell cycle effects of DREAM and MMB are of particular interest in the context of HGSOC (62). HGSOC is the most lethal of the gynecologic malignancies that is typically diagnosed at an advanced stage, with a median survival rate <5 years (63, 64). The majority of patients treated with surgery and platinum-based chemotherapy have a complete response to therapy, while 25% patients have primary platinum resistance associated with decreased survival (65). While long disease-free intervals are common, they typically shorten over time, and patients become platinum resistant (66). HGSOC tumors are characterized by loss-of-function p53 mutations, making it plausible that the inability to assemble DREAM and enter quiescence could contribute to the initial high treatment sensitivity of HGSOC. It is important to investigate the status of key cell cycle regulators, including DREAM and MMB, in HGSOC with primary and acquired platinum resistance.

Ovarian cancer recurrence has been linked to the formation of cellular aggregates (spheroids) composed of quiescent cells and disseminated through peritoneal fluid. The DREAM complex is assembled upon spheroid formation and plays an active role in maintaining quiescence (67). Inactivation of DREAM by depleting DYRK1A or LIN52 in the ascites-derived HGSOC primary cell lines resulted in reduced spheroid cell viability upon carboplatin treatment. DREAM inactivation led to enhanced cell death. Similarly, DYRK1A inhibition with small molecule drug INDY led to MMB complex formation, compromised DREAM-mediated cell cycle gene repression, and enhanced cell death in HGSOC primary cultures in response to carboplatin treatment (67, 68). This result provides rationale for investigating the therapeutic potential of targeting DREAM in combination with cytotoxic chemotherapy. Pharmacological inhibition of DYRK1A is currently under consideration for the treatment of conditions in which it is overexpressed (Down syndrome and Alzheimer disease) as well as Down syndrome-associated pediatric leukemia (69, 70). Several specific and efficient DYRK1A inhibitors have been reported but further studies are needed to identify candidates suitable for clinical use. The plant-derived alkaloid drug harmine is an effective inhibitor of DYRK1A, but its clinical utility is limited by its potent monoamine oxidase A inhibitory activity (61, 70, 71). A recent report describes a clinically safe and potent new DYRK1A inhibitor CX-4549 that is active against several DYRK1A substrates in cell- and animal-based assays (72). Its ability to block DREAM assembly and entry into quiescence has not yet been evaluated.

Pharmacologically targeting DYRK1A could be challenging because this ubiquitously expressed kinase is involved in various processes in different cell types. Some cancers express high levels of DYRK1B, a close homolog present mostly in skeletal muscle. Similar to DYRK1A, DYRK1B also phosphorylates S28 in LIN52 and stabilizes DREAM (10). DYRK1B inhibition was thus proposed as a way to circumvent the untoward effects of DYRK1A pharmacological inhibitors (73, 74). Several studies suggest that tumor cells expressing DYRK1B more heavily rely on its activity and that DYRK1B depletion compromises the ability to maintain quiescence (75–78). Notably, DYRK1B protein expression is detected in 75% of resected ovarian tumors and up to 10% of ovarian cancers have *DYRK1B* gene amplification (19, 77, 79). Treatment of the ovarian cancer cells overexpressing DYRK1B with RO5454948 (inhibitor of both DYRK1 kinases) resulted in cell cycle re-entry and apoptosis, whereas the normal ovarian epithelial cells remained viable (78). However, the only known drug with some selectivity against DYRK1B (fivefold higher potency than for DYRK1A *in vitro*), AZ191, has not been evaluated *in vivo* (80).

## Conclusion

Overall, the dual role of MuvB in both cellular quiescence and proliferation highlights the intricacy of cell cycle control as well as the importance of cooperation between tumor suppressor pathways. While MMB function is tied to aggressive disease and poor prognosis in cancer, there is robust evidence implicating DREAM function in chemotherapy resistance and cancer cell survival. Therefore, a shift in the utilization of MuvB, for either DREAM or MMB formation, could represent a strategy by which cancer cells exploit the cell cycle. Manipulating MuvB could provide substantial regulatory control over the cell cycle, as supported by evidence that both DREAM (*via* blocking DYRK1 kinases) and MMB (*via* druggable downstream targets) could be targeted for cancer therapy. Given the ongoing development of clinically viable drugs, the next challenge will be to determine optimal conditions for applying these treatments. Further structure–function studies of the DREAM and MMB, as well as their regulatory signaling pathways, will inform treatment strategies for targeting specific states of MuvB—either inhibiting cell proliferation or entry into quiescence. Although MuvB has been explored at the cellular level, studies with patient samples and clinical data are needed to validate *in vitro* findings and develop the personalized treatments required to modulate the cell cycle key, MuvB.

## Author Contributions

AI performed the literature searches, drafted figures, and wrote the manuscript. LL reviewed the concept, prepared figures, and wrote the manuscript.

## Conflict of Interest Statement

The authors declare that the research was conducted in the absence of any commercial or financial relationships that could be construed as a potential conflict of interest.
